# Association Between Genetic Polymorphisms of Metabolic Enzymes and Azathioprine-Induced Myelosuppression in 1,419 Chinese Patients: A Retrospective Study

**DOI:** 10.3389/fphar.2021.672769

**Published:** 2021-05-18

**Authors:** Zhao-Yang Chen, Yang-Hui Zhu, Ling-Yan Zhou, Wei-Qiao Shi, Zhou Qin, Bin Wu, Yu Yan, Yu-Wen Pei, Ning-Ning Chao, Rui Zhang, Mi-Ye Wang, Ze-Hao Su, Xiao-Jun Lu, Zhi-Yao He, Ting Xu

**Affiliations:** ^1^ Department of Pharmacy, State Key Laboratory of Biotherapy and Cancer Center, Med-X Center for Informatics, National Clinical Research Center for Geriatrics, West China Hospital, Sichuan University, Chengdu, China; ^2^ Institute of Respiratory Health, Frontiers Science Center for Disease-related Molecular Network, West China Hospital, Sichuan University, Chengdu, China; ^3^ Department of Information Center, Engineering Research Center of Medical Information Technology of the Education Ministry, West China Hospital, Sichuan University, Chengdu, China; ^4^ Med-X Center for Informatics, West China Hospital, Sichuan University, Chengdu, China; ^5^ Department of Laboratory Medicine, West China Hospital, Sichuan University, Chengdu, China; ^6^ Key Laboratory of Drug-Targeting and Drug Delivery System of the Education Ministry, Sichuan Engineering Laboratory for Plant-Sourced Drug and Sichuan Research Center for Drug Precision Industrial Technology, West China School of Pharmacy, Sichuan University, Chengdu, China

**Keywords:** azathioprine, ITPA, TPMT, NUDT15, myelosuppression, adverse drug reaction

## Abstract

The aim of this study was to investigate the correlation between genetic polymorphisms of azathioprine-metabolizing enzymes and adverse reactions of myelosuppression. To this end, a retrospective analysis was performed on 1,419 Chinese patients involving 40 different diseases and 3 genes: *ITPA* (94C>A), TPMT*3 (T>C), and *NUDT15* (415C>T). Strict inclusion and exclusion criteria were established to collect the relative cases, and the correlation between azathioprine and myelosuppression was evaluated by adverse drug reaction criteria. The mutation rates of the three genes were 29.32, 3.73, and 21.92% and grades I to IV myelosuppression occurred in 54 (9.28%) of the 582 patients who took azathioprine. The highest proportion of myelosuppression was observed in 5 of the 6 (83.33%) patients carrying the *NUDT15* (415C>T) TT genotype and 12 of the 102 (11.76%) patients carrying the *NUDT15* (415C>T) CT genotype. Only the *NUDT15* (415C>T) polymorphism was found to be associated with the adverse effects of azathioprine-induced myelosuppression (odds ratio [OR], 51.818; 95% CI, 5.280–508.556; *p* = 0.001), which suggested that the *NUDT15* (415C>T) polymorphism could be an influencing factor of azathioprine-induced myelosuppression in the Chinese population. Epistatic interactions between *ITPA* (94C>A) and *NUDT15* (415C>T) affect the occurrence of myelosuppression. Thus, it is recommended that the genotype of *NUDT15* (415C>T) and *ITPA* (94C>A) be checked before administration, and azathioprine should be avoided in patients carrying a homozygous *NUDT15* (415C>T) mutation. This study is the first to investigate the association between genetic polymorphisms of these three azathioprine-metabolizing enzymes and myelosuppression in a large number of cases with a diverse range of diseases.

## Introduction

Azathioprine (AZA) is a classic immunosuppressant that is widely used for post-transplant rejection, severe rheumatoid arthritis, systemic lupus erythematosus, pemphigus (Joly et al., 2020), inflammatory bowel disease (Ran et al., 2020), dermatomyositis, and other diseases (Mack et al., 2020). It is also recommended for the treatment of immune checkpoint inhibitor-related renal and musculoskeletal adverse events (Thompson et al., 2020). However, AZA can also cause drug adverse reactions (ADRs), including myelosuppression, hepatotoxicity, gastrointestinal reactions (nausea, vomiting, and diarrhea), and alopecia (Food and Drug Administration, 2018). Among them, myelosuppression is particularly harmful and could result in leukopenia, thrombocytopenia, pancytopenia, and even some life-threatening conditions (Panda et al., 2018).

AZA is metabolized into the active 6-thioguanine nucleotides (6-TNGs) by a series of enzymes *in vivo* (Yang et al., 2014; Moon and Loftus, 2016; Kishibe et al., 2018; Wang et al., 2018). AZA is first metabolized to 6-mercaptopurine (6-MP) by glutathione S-transferase (GST), and then converted into 6-thioinosine monophosphate (6-TIMP) with the help of hypoxanthine-guanine phosphoribosyl transferase (HGPRT). Subsequently, 6-TIMP is dehydrogenized into 6-thioxanthosine monophosphate (6-TXMP) by inosine monophosphate dehydrogenase (IMPDH), and then further metabolized to 6-TNGs by guanosine monophosphate synthetase (GMPS), which finally integrates into DNA and RNA molecules to exert cytotoxic and immunosuppressive effects. Moreover, 6-TGTP also binds to Rac1, and inactivates it by regulating the Vav-Rac1 signaling pathway in T lymphocytes; this results in the inhibition of Rac1 target genes, such as nuclear factor kappa beta (NF-κβ), finally leading to the increased apoptosis of activated T lymphocytes (Tiede et al., 2003; Poppe et al., 2006) (Supplementary Figure S1).

To reduce the risk of ADRs resulting from the use of AZA, researchers have attempted to establish AZA-metabolizing enzymes to predict the occurrence of myelosuppression and liver toxicity and adjust the dosage according to the genotype. The Clinical Pharmacogenetics Implementation Consortium (CPIC) first published guidelines for adjusting the dose of AZA based on the thiopurine S-methyltransferase (*TPMT*) polymorphism in 2011 (Relling et al., 2011), which were later updated in 2013 and 2018 (Relling et al., 2013; Relling et al., 2019). Currently, the guidelines recommend that patients with a normal *TPMT* metabolizer can use the standard recommended dose, those with intermediate metabolizers are recommended to use 30–80% of the normal dose, and those with poor metabolizers with nonmalignant conditions are not recommended to use AZA. Patients with poor *TPMT* metabolizers with malignancy are recommended to reduce the daily dose by 10-fold and to receive the dose thrice weekly instead of daily. Although some previous studies have investigated the association of AZA-induced ADRs with *TPMT*, inosine triphosphate pyrophosphatase (*ITPA*), nucleoside diphosphate-liked moiety X motif 15 (*NUDT15*), *GST,* multidrug resistance protein 4 (*MRP4*), *HGPRT*, *IMPDH*, and xanthine oxidase (*XO*), the results were varying due to ethnic differences in gene distribution (Krishnamurthy et al., 2008; Kudo et al., 2009; Yang et al., 2014; Burgis, 2016; Choi et al., 2019; Yang et al., 2019). Moreover, the dosage recommended in the CPIC guidelines is inaccurate in that it cannot be individualized among individuals of different races and regions. Among these genes, the most well-studied are *ITPA*, *TPMT*, and *NUDT15*. Some studies have reported that mutations in *ITPA* have no association with AZA-induced myelosuppression (Al-Judaibi et al., 2016; Steponaitiene et al., 2016). Other studies have indicated that the incidence of hepatotoxicity increases with a high *TPMT* enzyme activity, and that there is a high risk of myelosuppression with a low *TPMT* enzyme activity, due to its homozygous mutation. The Food and Drug Administration (FDA) recommends that the *TPMT* genotype of patients should be determined before using AZA (FDA). However, studies have shown that the frequency of *TPMT* gene mutations in the Asian population is only approximately 1.5–3%, thereby showing a high specificity but a low sensitivity. However, Asians have a low tolerance to AZA and a high incidence of leukopenia, which makes it necessary to explore predictive genes suitable for the Asian population specifically. In recent years, some studies have shown that *NUDT15* might be highly correlated with AZA-induced myelosuppression in Asians (Chao et al., 2017; Wang et al., 2018; Banerjee et al., 2020; Kang et al., 2020), and the CPIP guideline also recommends that the *NUDT15* genotype should be determined prior to the administration of AZA (2018) (Relling et al., 2019).

AZA is widely used in clinical settings, and genetic testing is essential for patients who need to take this drug for an extended duration. The abovementioned genes, *ITPA*, *TPMT*, and *NUDT15*, are currently being tested at the West China Hospital of Sichuan University, and the dose of AZA is being adjusted by doctors in accordance with the results of genetic tests to avoid adverse reactions. However, it has been found clinically that some patients with no mutations in these genes suffered myelosuppression, while others with homozygous mutations did not. To provide a reference for the analysis of genetic test results and accurate medication, this study was performed to explore the correlation between the polymorphism of these three genes and AZA-induced myelosuppression. As large-volume analytical studies, especially those involving diverse diseases, remain rare, this study is particularly important given that we examined a large number of cases with various diseases.

## Materials and Methods

### Patients

All included cases were collected from outpatient, emergency, and inpatient data of the West China Hospital of Sichuan University.

### Inclusion and Exclusion Criteria

Related data of patients who underwent genetic testing of AZA-metabolizing enzyme genes from January 2016 to January 2019 in our hospital were extracted from the database. After the removal of duplicates, the patient information, including age, sex, clinical department, diagnosis, white blood cell count (WBC), and AZA daily dose, was compiled using the hospital information system. To determine myelosuppression, patients taking AZA who had complete routine blood examination results were included, while those who did not receive AZA or had incomplete WBC records were excluded.

### Myelosuppression Criteria

According to the Common Terminology Criteria for Adverse Events (CTCAEs) version 5.0 published by the United States Department of Health and Human Services and the hospital leukocyte count index standard, myelosuppression was defined as a WBC count <3.5 × 10^9^/l; a WBC count of 3–3.5 × 10^9^/l was defined as grade I, 2–3 × 10^9^/l as grade II, 1–2 × 10^9^/l as grade III, and <1 × 10^9^/l as grade IV. Adverse drug reaction correlation evaluation criteria of the National Medical Products Administration of China were used to evaluate AZA and myelosuppression correlation, and the Naranjo score was also used when the judgment results were controversial (National Health Commission PRC, 2011; Naranjo et al., 1981). The result “possible” was considered to be an adverse reaction of myelosuppression, and the results were judged by two clinical pharmacists after a double cross-check.

### Statistical Analysis

Microsoft Office Excel 2010 was used to input data, and SPSS 25.0 (IBM Corp., Armonk, NY, United States) was used for statistical analysis. Continuous variables are presented as mean ± SD. The independent t-test or Mann–Whitney U test was used to investigate the difference between two unrelated groups, and the one-way analysis of variance was used for comparison between multiple groups. Categorical variables were compared by the chi-square test or Fisher's test, and the Bonferroni correction was used for pairwise comparison between groups. The related factors of myelosuppression were analyzed by logistic regression analysis. Chi-square goodness-of-fit was used to confirm the agreement of the *ITPA* (94C>A), TPMT*3 (T>C), and *NUDT15* (415C>T) genotype frequencies with the expected frequencies (Hardy–Weinberg equilibrium). The multifactor dimensionality reduction (MDR) method was used to examine gene–gene interactions, and MDR Permutation Testing software (version 1.0 Beta 2) was used for replacement testing. *P*-values < 0.05 were considered statistically significant.

## Results

### Patient Characteristics

**FIGURE 1:**
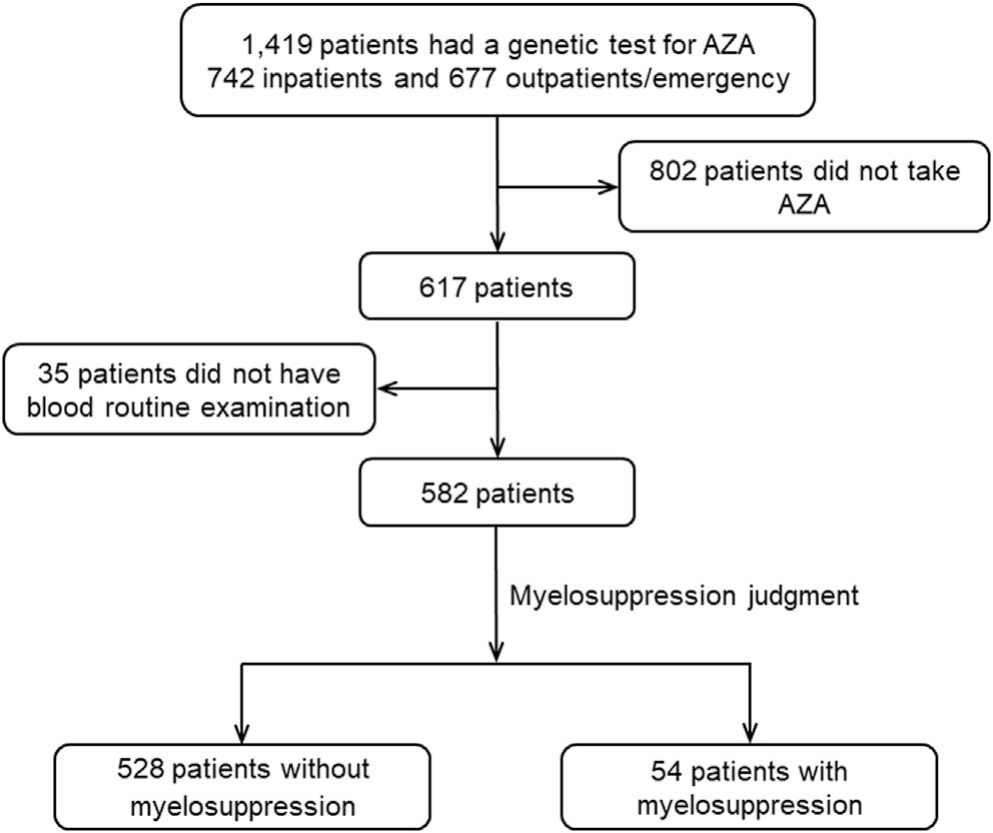
Flow diagram for the inclusion and exclusion of patients.

A total of 1,419 available cases were covered in this study, including 742 (52.29%) inpatients and 677 (47.71%) outpatients/emergency patients (Figure 1). Among them, there were more female patients (65.19%), and the average age was 45.96 ± 14.41 years. Of the total cases, males comprised 494 (34.81%), with an average age of 42.74 ± 16.32 years. Nineteen departments were involved in the study, and the study population included 40 diseases, including pemphigus, inflammatory bowel disease, and autoimmune hepatitis (Supplementary Figure S2), among which, pemphigus was the most common (347, 24.45%).

### Genetic Mutation

Among the 1,419 patients, 1,279 (90.13%) had *ITPA* (94C>A) (rs1127354), TPMT*3 (T>C) (rs1142345), and *NUDT15* (415C>T) (rs116855232), while 140 (9.87%) had TPMT*3 (T>C) and *NUDT15* (415C>T). The genotypes of all patients are shown in Supplementary Table S1. The *ITPA* (94C>A), TPMT*3 (T>C), and *NUDT15* (415C>T) genotype distributions were in Hardy–Weinberg equilibrium (*p* = 0.959, 0.811, and 0.406, respectively). The mutation rate of TPMT*3 (T>C) was the lowest (3.73%), similar to the previous reports in Asian populations (Kumagai et al., 2001; Chen et al., 2014; van et al., 2014). The respective proportions of wild type, heterozygous mutation, and homozygous mutation of the three genes were statistically significant (*p* = 1.374 × 10^−68^), and pairwise comparison between different genotypes by chi-square test also showed statistical significance. The data are shown in Table 1.

**TABLE 1:** Mutations of *ITPA*, TPMT*3, and *NUDT15*.

Genotype	*ITPA* (94C>A)	TPMT*3 (T>C)	*NUDT15* (415C>T)	*P*-value^a^		
Wild type	904 (70.68%)	1,366 (96.27%)	1,108 (78.08%)	1.551 × 10^−72^	0.059 × 10^−3^	1.868 × 10^−46^
Heterozygote	340 (26.58%)	51 (3.59%)	283 (19.95%)			
Homozygote	35 (2.74%)	2 (0.14%)	28 (1.97%)			
Total	1,279	1,419	1,419			

Data are n (%).

^a^ The differences in mutations were statistically significant between ITPA (94C>A) and TPMT*3 (T>C) (p = 1.551 × 10^−72^), and between ITPA (94C>A) and NUDT15 (415C>T) (*p* = 0.059 × 10^−3^), and between TPMT*3 (T>C) and NUDT15 (415C>T) (*p* = 1.868 × 10^−46^).

### Overall Incidence of Myelosuppression

A total of 617 (43.48%) patients had AZA administration records, 582 (94.33%) of whom had complete routine blood examination results. Myelosuppression occurred in 54 (9.28%) patients, and the incidence of myelosuppression with grades from I to IV was 37.04% (20/54), 42.59% (23/54), 11.11% (6/54), and 9.26% (5/54), respectively.

### Incidence of Myelosuppression According to Genotype

#### *ITPA* (94C>A) Genotype

A total of 516 patients carrying the *ITPA* (94C>A) genotype were included, among whom, 48 (9.30%) suffered from myelosuppression (grades I-IV). Patients carrying the *ITPA* (94C>A) AA genotype had the highest risk of myelosuppression, with an incidence of 25.00%, although this was not statistically significant. The mean daily dose (MDD) of AZA was significantly different among patients with different genotypes (*p* = 0.002). Multiple comparison results showed that only the AZA doses between the AC and CC genotype were significantly different (*p* = 0.323 × 10^−3^) (Supplementary Table S2).

#### TPMT*3 (T>C) Genotype

A total of 582 cases with the TPMT*3 (T>C) genotype were included, among whom, 54 (9.28%) had ADRs of myelosuppression (grades I-IV). Similarly, the incidence of myelosuppression varied according to genotype; patients carrying the TPMT*3 (T>C) TC heterozygous mutation had the highest rate (20.00%), but there were no significant differences among the three groups. There were also no significant differences among the MDD of AZA among the different genotypes (Supplementary Table S2).

#### *NUDT15* (415C>T) Genotype

The number of patients carrying *NUDT15* (415C>T) gene was 582, 54 (9.28%) of whom had ADRs of myelosuppression (grades I-IV). The incidence of myelosuppression varied by genotype; patients carrying the *NUDT15* (415C>T) TT homozygote mutant had the highest rate (83.33%; 3 [60.00%] with grade IV), and the difference among the three genotypes was significant (*p* = 0.008 × 10^−3^). After the Bonferroni correction, there was a statistically significant difference in the myelosuppression rate between patients with the TT genotype and the other two genotypes (CT and CC) (TT, CC: *p* = 7.707 × 10^−11^ and TT, CT: *p* = 0.003 × 10^−3^), and there was no statistically significant difference between the CT and CC genotypes (*p* = 0.194). The WBC count was significantly different among the three genotypes (*p* = 0.002), but the results of multiple comparisons showed that only the TT and CC genotypes were significantly different (*p* = 0.002). The difference in the MDD of AZA among different genotypes was also significant (*p* = 0.010), but the results of multiple comparisons showed that it was only statistically significant between the CT and CC genotypes (*p* = 0.003) (Supplementary Table S2).

### Myelosuppression According to Genotype Combinations

Our results show that all of the patients who carried the *NUDT15* (415C>T) TT homozygote mutation, regardless the genotypes of TPMT*3 (T>C) and *ITPA* (94C>A), had a higher incidence of myelosuppression, and mostly at grade IV. Moreover, 20 of 313 (6.39%) patients carried the wild-type versions of these three genes, one of whom had grade IV myelosuppression. One patient carried a homozygote mutation of TPMT*3 (T>C), with wild-type *NUDT15* (415C>T) and *ITPA* (94C>A), and did not suffer myelosuppression (Table 2).

**TABLE 2:** Incidence of myelosuppression in patients with different genotype combinations.

*ITPA* (94C>A)	TPMT*3 (T>C)	*NUDT15* (415C>T)	Myelosuppression	Grade (n)
CA	TT	TT	1/1 (100%)	I (1)
-	TC	TT	1/1 (100%)	IV (1)
CC	TT	TT	3/4 (75.00%)	IV (2), I (1)
CA	TC	CC	2/5 (40.00%)	II (2)
AA	TT	CC	2/7 (28.57%)	II (1), III (1)
-	TT	CT	2/14 (14.29%)	I (1), III (1)
CC	TT	CT	8/59 (13.56%)	I (2), II (5), III (1)
CA	TT	CC	9/87 (10.34%)	I (6), II (2), III (1)
CC	TC	CC	1/10 (10.00%)	III (1)
CA	TT	CT	2/26 (7.69%)	II (1), IV (1)
CC	TT	CC	20/313 (6.39%)	I (8), II (10), III (1), IV (1)
-	TT	CC	3/49 (6.12%)	I (1), II (2)
AA	TT	CT	0/1	–
CC	TC	CT	0/2	–
-	TC	CC	0/2	–
CC	CC	CC	0/1	–

### Factors Associated With Myelosuppression

Based on the occurrence of myelosuppression, all of the 582 patients who received AZA treatment and had routine blood examinations were divided into two groups: group A suffered myelosuppression and group B did not. The results showed that there were more females in both groups, but there was no significant difference in the ratio of males to females (*p* = 0.240), ages (*p* = 0.866), nor the MDD of AZA (*p* = 0.410) between the two groups of patients. The number of patients carrying different genotypes of *ITPA* (94C>A) and TPMT*3 (T>C) was also not significantly different between the two groups, although the number of patients carrying *NUDT15* (415C>T) mutations was significantly different (*p* = 0.008 × 10^−3^) (Table 3).

**TABLE 3:** Comparison of differences between groups with and without myelosuppression.

	Group A (*n* = 54)	Group B (*n* = 528)	*P*-value
Gender: male/female	15/39	189/339	0.240
Age	43.50 ± 14.27	43.15 ± 14.42	0.866
*ITPA* (94C>A) CC/CA/AA	32/14/2	357/105/6	0.155
TPMT*3 (T>C) TT/TC/CC	50/4/0	511/16/1	0.188
*NUDT15* (415C>T) CC/CT/TT	37/12/5	437/90/1	0.008 × 10^−3^
MDD of AZA (mg)	72.21 ± 35.44	68.90 ± 27.27	0.410

Data are n (%) or mean ± SD.

Binary logistic regression analysis of myelosuppression-related factors showed that the risk of myelosuppression was significantly higher in patients with an *NUDT15* (415C > T) TT genotype than in those with the wild type (odds ratio [OR], 51.818; 95% CI, 5.280–508.556; *p* = 0.001). Beyond this, no significant difference in the incidence of myelosuppression was found among the patients with other genotypes of these three genes compared with their corresponding wild type. Other factors, including age (*p* = 0.722), sex (*p* = 0.075), and dose (*p* = 0.490), had no significant association with the incidence of myelosuppression (Table 4).

**TABLE 4:** Regression analysis of factors associated with myelosuppression.

Variable	*P*-value	OR	95% CI
Gender (referent: male)	0.722	0.996	0.974–1.018
Age	0.075	1.940	0.934–4.028
MDD of azathioprine (mg)	0.490	1.004	0.993–1.015
*ITPA* (94C>A) CA (referent: CC)	0.197	1.585	0.787–3.191
*ITPA* (94C>A) AA (referent: CC)	0.061	4.945	0.928–26.358
TPMT*3 (T>C) TC (referent: TT)	0.185	2.420	0.655–8.946
TPMT*3 (T>C) CC (referent: TT)	1.000	0.000	0.000–
*NUDT15* (415C>T) CT (referent: CC)	0.216	1.624	0.754–3.497
*NUDT15* (415C>T) TT (referent: CC)	0.001	51.818	5.280–508.556

OR: odds ratio, CI: confidence interval.

### Gene–Gene Interactions

The gene–gene interaction results are shown in Table 5. The *NUDT15* (415C>T) locus had the highest testing balanced accuracy among the 3 SNPs. The optimal interaction models include *NUDT15* (415C>T) and *ITPA* (94C>A) with a maximum cross-validation (CV) consistency of 10 out of 10 and a maximum testing balanced accuracy of 0.5921 (*p* < 0.05 on the basis of 1000-fold permutation testing).

**TABLE 5:** Epistatic interactions between the variants of azathioprine metabolism influencing myelosuppression.

Model	Training balance accuracy	Testing balance accuracy	Cross-validation Consistency	χ^2^ (*P*)	OR (95%CI)
*NUDT15* (415C>T)	0.5732	0.4969	8/10	6.58 (0.0103)	2.21 (1.19–4.09)
*NUDT15* (415C>T) and *ITPA* (94C>A)	0.6157	0.5921	10/10	12.20 (0.0005)	2.66 (1.51–4.69)
*NUDT15* (415C>T), *ITPA* (94C>A), and TPMT*3 (T>C)	0.6199	0.5507	10/10	12.47 (0.0004)	2.69 (1.53–4.74)

OR: odds ratio, CI: confidence interval.

## Discussion

The retrospective case analysis was performed on a large number of relative cases involving various diseases. Current reports are mostly limited to a certain disease or a type [such as inflammatory bowel disease (Al-Judaibi et al., 2016; Wang et al., 2018; Walker et al., 2019; Kang et al., 2020), autoimmune disease (Fei et al., 2018; Fan et al., 2019), and acute lymphoblastic leukemia (Yang et al., 2015; Zhu et al., 2018)], and have generally included a small number of study subjects, with a focus on only one or two genes. In this study, 40 diseases were included, which covered all of the indications for AZA, and the correlation between genetic polymorphisms of AZA-metabolizing enzymes and AZA-induced myelosuppression were compared under different pathological states.

The most common single-nucleotide polymorphism (SNP) loci of *ITPA* in the population are 94C>A and IVS2 + 21A>C, and the mutation of *ITPA* (94C>A) leads to a high risk of ADRs (Arenas et al., 2007). Mutations at this locus affect protein expression by reducing the expression of the full length transcript, decreasing the catalytic activity and stability, and altering mRNA splicing events such as missplicing of exons 2 and 3. Finally, these mutations result in a poor expression of an unstable, catalytically compromised protein, and affect the activity of ITPA (Burgis, 2016). The reported frequency of the *ITPA* (94C>A) A allele is higher in Asians (11–19%) than in Caucasian, Hispanics, and Africans (1–7%) (Maeda et al., 2005; Hawwa et al., 2008; Okada et al., 2009). In this study, the mutation rate of *ITPA* (94C > A) was 29.32%, and the frequency of carrying the *ITPA* (94C>A) A allele was 16.02%, which was consistent with that reported in the current Asian population. Odahara et al. reported that the mutation rate of this gene was 39.6% in 48 Japanese inflammatory bowel disease (IBD) patients, and that the incidence of leukopenia in patients carrying this mutation was 36.8% (Odahara et al., 2015). However, the study indicated that leukopenia cannot be clearly attributed to the *ITPA* (94C>A) mutation as there may be other influencing factors. Moreover, Wroblova et al. reported a 13.8% mutation rate of the *ITPA* (94C > A) gene in 188 IBD patients, but no confirmed association was found between its polymorphism and myelosuppression toxicity (Wroblova et al., 2012). These studies also indicated that *ITPA* genetic polymorphisms may be associated with influenza-like symptoms, arthralgia, and pancreatitis (Zabala-Fernandez et al., 2011; Wroblova et al., 2012). However, the sample sizes of previous studies have been limited, and relatively new studies are lacking. The number of patients included in our study is large, and the result is representative of the Chinese population. In our study, the incidence of myelosuppression in patients with different *ITPA* (94C>A) genotypes, from high to low, were homozygous mutation, heterozygous mutation, and wild type. Nevertheless, there was no significant difference in the incidence of myelosuppression among these three genotypes. Moreover, a correlation factor analysis suggested that compared to the wild type, homozygous, and heterozygous mutations in patients carried a high risk of myelosuppression, although this was not significant. Therefore, there was no significant correlation between the *ITPA* (94C>A) gene polymorphism and myelosuppression.

Collie-Duguid et al. reported that the rate of *TPMT* gene mutation was 10.1% (20/199) in Caucasians, 2.0% (2/99) in Southwest Asians, and 4.7% (9/192) in Chinese (Collie-Duguid et al., 1999). Common mutation loci in *TPMT* include TPMT*2, TPMT*3A, TPMT*3B, and TPMT*3C. TPMT*3A is the dominant locus in Caucasians, and TPMT*3C is the most common locus in Southeast Asian, African, and African–American populations. *TPMT* has ten exons, eight of which encode the 28-kDa protein. Nucleotide transversion (G238C) at one locus of TPMT*2 leads to the substitution of a rigid proline for a more flexible alanine residue at codon 80 (Krynetski et al., 1995). This mutation causes changes in the tertiary structure of the *TPMT* protein, which reduces the stability and catalytic ability of the protein. TPMT*3A contains two single nucleotide transversions, G460A in exon 7 and A719G in exon 10, which leads to amino acid substitutions at codon 154 (Ala > Thr) and codon 240 (Tyr > Cys) (Sahasranaman et al., 2008). TPMT*3B and TPMT*3C both have only one mutation locus, G460A in exon 7 and A719G in exon 10, respectively (Zelinkova et al., 2006). These variants destabilize the *TPMT* protein, and reduce its binding affinity to 6-MP (Naushad et al., 2021). In our study, the mutation rate of TPMT*3 (T>C) was the lowest, at 3.73%, which was close to the previously reported mutation rate of 2.90% (15/522) in the Japanese population (Kumagai et al., 2001), and 3.17% (4/126) and 4.60% (4/87) in the Chinese population (Chen et al., 2014; Fei et al., 2018). Moreover, in these two studies (Chen et al., 2014; Fei et al., 2018), all of the mutant genotypes of *TPMT* were heterozygous and no homozygous mutation was found. The higher number of participants in our study could better reflect the mutation rate of this gene in the Chinese population (low). Chen et al. suggested that the *TPMT* gene polymorphism in Chinese SLE patients had a low sensitivity to predict leukopenia, resulting in a limited clinical value; therefore, they recommended that the AZA dose should be adjusted by monitoring the enzyme activity of *TPMT* (Chen et al., 2014). Two other studies on Chinese patients with autoimmune diseases demonstrated that the polymorphism of *TPMT* was not clearly associated with AZA-induced leukopenia (Fei et al., 2018; Fan et al., 2019). Although a meta-analysis of 14 published studies, involving 2276 patients with IBD, showed an association between the *TPMT* polymorphism and AZA-induced myelosuppression in Caucasians (*p* < 0.00001; pooled OR, 6.97; 95% CI, 3.89–12.47), no significant correlation was found in Asians (*p* = 0.12) (Liu et al., 2015). In our study, only two patients carried the TPMT*3 (T>C) CC genotype, one of whom had an AZA treatment history but no myelosuppression. The incidence of myelosuppression in patients with the TPMT*3 (T>C) TC genotype was significantly higher than that in patients with the wild genotype, but the difference was not statistically significant. The correlation factor analysis showed that patients with TPMT*3 (T>C) TC had a higher risk of myelosuppression (OR, 2.420; 95% CI, 0.655–8.946) those with the wild type, but the difference was not significant (*p* = 0.185). Therefore, there was no correlation between the polymorphism of TPMT*3 (T>C) and myelosuppression. Some studies in Western countries demonstrated a correlation between the *TPMT* gene polymorphism and the ADR of blood toxicity (Zabala-Fernandez et al., 2011; Al-Judaibi et al., 2016; Steponaitiene et al., 2016); however, for the Asian population, especially Chinese, there was no significant association between the *TPMT* gene polymorphism and myelosuppression.

There are four common mutation loci of *NUDT15*, including rs116855232, rs554405994, rs186364861, and rs147390019 (Moriyama et al., 2016), the most common of which is rs116855232 (415C>T, protein sequence p.Arg139Cys). Studies have reported that the *NUDT15* (415C>T) mutation does not affect enzymatic activity but does adversely affect protein stability (Valerie et al., 2016). This may be due to the loss of supportive intramolecular bonds, leading to a rapid degradation of proteasomes in cells. Other reports noted that *NUDT15* variants have no impact on the binding of “dGTP” to the *NUDT* protein. The *NUDT15* (415C>T) mutation increases aggregation “hot spots” and induces unfavorable torsion in the protein (Naushad et al., 2021). The frequency of mutation for this locus (15–30%) is higher in East Asian populations, including Japanese (Kakuta et al., 2018; Tanaka et al., 2018), Chinese (Chao et al., 2017; Fei et al., 2018), Koreans (Kim et al., 2017; Yi et al., 2018), and Indians (Banerjee et al., 2020), while the mutation rate is low in Caucasians (Yang et al., 2015; Walker et al., 2019) (European: 0.5–0.8% and Hispanic: 7.7%). Some studies have investigated the mutation rate of *NUDT15* (415C>T) in the Chinese population, but the majority have had small sample sizes. Fan et al. reported an *NUDT15* (415C>T) mutation rate of 17.45% (26/149) in Chinese patients with autoimmune hepatitis, among whom, only 2 patients (1.34%) had homozygous mutations (Fan et al., 2019). Fei et al. studied 87 Chinese patients with autoimmune diseases, and found an *NUDT15* (415C>T) mutation rate of 32.18%, with only one patient (1.15%) carrying a homozygous mutation (Fei et al., 2018). In the current study, the *NUDT15* (415C>T) mutation rate was 21.92%, and 28 (1.97%) patients had an *NUDT15* (415C>T) homozygous mutation; these results are higher than those reported by Fan et al. but lower than those of Fei et al. In addition, Kakuta and colleagues found a 25.27% mutation rate of this gene among 1,282 Japanese patients (Kakuta et al., 2018), which was close to the 21.92% observed in our study. Therefore, it is conceivable that our data truly reflect the mutation rate of *NUDT15* in the Chinese population. Current studies suggest that polymorphism of *NUDT15* is significantly associated with leukopenia or myelosuppression (Moriyama et al., 2016; Chao et al., 2017; Kim et al., 2017; Fei et al., 2018; Kakuta et al., 2018; Wang et al., 2018; Fan et al., 2019; Kang et al., 2020). Moreover, the risk of adverse reactions has been found to be much higher in people carrying homozygous mutations than in those with the wild genotype. Our results showed that the rate of AZA-induced myelosuppression in patients carrying the *NUDT15* (415C>T) TT genotype was as high as 83.33%, and the incidence of grade IV myelosuppression was 60.00%, while patients with a heterozygous mutation and wild type had rates of 11.76 and 7.81%, respectively. Moreover, the incidence of myelosuppression was significantly different among patients with homozygous mutations, heterozygous mutations, and wild type (*p* = 0.008 × 10^−3^). Given that there was no significant difference in the MDD of AZA among these patient groups, the interference of dose difference on the incidence of myelosuppression could be eliminated. The correlation factor analysis showed that compared to the wild type, people carrying the homozygous mutant genotype had an extremely high risk of myelosuppression (OR, 51.818; 95% CI, 5.280–508.556; *p* = 0.001). The mutation frequency of *NUDT15* (415C>T) was 21.92%, which was significantly higher than the 3.73% of TPMT*3 (T>C) (*p* = 1.868 × 10^−46^). Additionally, the analysis of factors associated with myelosuppression showed that the polymorphism of *NUDT15* (415C>T) was significantly associated with myelosuppression; thus, the *NUDT15* (415C>T) polymorphism is a promising predictor of AZA-induced myelosuppression in the Chinese population. According to the results, it is recommended to test the genotype of *NUDT15* (415C>T) before taking AZA, and AZA should be avoided in patients with a homozygous mutant genotype.

In the current study, the overall incidence of myelosuppression was 9.28%, which was close to the 8.05% (12/149) and 8.7% (81/935) described previously in Chinese and Indian populations (Fan et al., 2019; Banerjee et al., 2020), but lower than 18.17–23.81% reported in the other studies mentioned above (Kim et al., 2017; Fei et al., 2018; Kakuta et al., 2018; Yang et al., 2019). This difference may be due to the inherent limitations of the retrospective study and incomplete information on medication and examination which may lead to the absence of myelosuppression records for some patients. In addition, within the 582 patients with medication records, 83 of patients received an adjusted dose of AZA according to the gene test results to the safe range. The analysis of myelosuppression-related factors showed that sex, age, the MDD of AZA, and polymorphisms of *ITPA* (94C>A) and TPMT*3 (T>C) had no significant association with myelosuppression, and that only the polymorphism of *NUDT15* (415C>T) was an influencing factor. The analysis of different combinations of genotypes indicate that patients with the *NUDT15* (415C>T) T allele were prone to suffer from myelosuppression and those who carried the *NUDT15* (415C>T) TT genotype faced an even high risk. The results of gene–gene interactions showed that *NUDT15* (415C>T) had the highest testing balanced accuracy, which also proved that this gene locus had a strong correlation with myelosuppression. There might be an interaction between *ITPA* (94C>A) and *NUDT15* (415C>T) loci, which together affected the occurrence of myelosuppression induced by azathioprine. At present, the epistatic interactions among the above three gene loci had not been reported. This study was the first to analyze the gene–gene interactions among *ITPA*, *NUDT15*, and *TPMT*. We also found that in patients whose three genes were the wild type, 20 (6.39%) of them suffered from myelosuppression, and one case was grade IV. This finding suggests that these genes are not sufficient to predict myelosuppression in all patients, and there may be other relative metabolic enzyme genes that remain to be explored in future studies (Inman et al., 2018).

This study has some limitations. First, the retrospective nature of the study meant that the information was incomplete in some cases, and some medication records and test results were missing; in particular, one patient with the TPMT*3 (T>C) CC genotype had no medication records, which resulted in the exclusion of this population. In addition, as the genotype detection of *ITPA* (94C>A) was only initiated in our hospital in the last few years, there were 140 cases in whom only TPMT*3 (T>C) and *NUDT15* (415C>T) were detected, with no information on the *ITPA* (94C>A) genotype. The degree of AZA-induced myelosuppression could only be evaluated by the information provided in the cases, which cannot be used to reconstruct the actual situation at the time, making it difficult to truly evaluate the severe grades of myelosuppression.

## Conclusion

Our findings suggest that the polymorphism of *NUDT15* (415C>T) is a significantly relative factor in the context of AZA-induced myelosuppression, and epistatic interactions between *ITPA* (94C>A) and *NUDT15* (415C>T) affect the occurrence of myelosuppression. Therefore, it is recommended to test these two genes prior to administration of AZA. In people carrying a homozygous mutation of *NUDT15* (415C>T), the risk of myelosuppression is very high, and therefore AZA should be avoided during their treatment. However, in our hospital, the cost of the detection of these three metabolic enzyme genes is 628 times that of one tablet of AZA (100 mg). Thus, the detection of *ITPA* (94C>A) and TPMT*3 (T>C) is not necessarily recommended for economic reasons but only to test the genotype of *NUDT15* (415C>T) for patients who have difficulty in paying medical expenses. Moreover, there may be other AZA-metabolizing enzyme genes that could better predict the incidence of AZA-induced myelosuppression, and further investigations are needed.

## Data Availability

All datasets presented in this study are included in the article/Supplementary Material.
