# Supplementary material for: Association Between Genetic Polymorphisms of Metabolic Enzymes and Azathioprine-Induced Myelosuppression in 1,419 Chinese Patients: A Retrospective Study
Source: Front Pharmacol. 2021 May 18;12:672769. doi: 10.3389/fphar.2021.672769 (PMC8167793; doi:10.3389/fphar.2021.672769)
Supplement: Supplementary file 1 [file Presentation1.pdf]

Supplementary materials

Association between genetic polymorphisms of metabolic enzymes and azathioprine-induced myelosuppression in 1,419 Chinese patients: a retrospective study

Zhao-Yang Chen[1,†], Yang-Hui Zhu[1,†], Ling-Yan Zhou[1], Wei-Qiao Shi[1], Zhou Qin[1], Bin Wu[1], Yu Yan[1], Yu-Wen Pei[1], Ning-Ning Chao[2], Rui Zhang[3], Mi-Ye Wang[3], Ze-Hao Su[4], Xiao-Jun Lu[5,*], Zhi-Yao He[1,6,*], Ting Xu[1,6]

[1] Department of Pharmacy, State Key Laboratory of Biotherapy and Cancer Center, Med-X Center for Informatics, National Clinical Research Center for Geriatrics, West China Hospital, Sichuan University, Chengdu, Sichuan 610041, China

[2] Institute of Respiratory Health, Frontiers Science Center for Disease-related Molecular Network, West China Hospital, Sichuan University, Chengdu, Sichuan 610041, China

[3] Department of Information Center, Engineering Research Center of Medical Information Technology of the Education Ministry, West China Hospital, Sichuan University, Chengdu, Sichuan 610041, China

[4] Med-X Center for Informatics, West China Hospital, Sichuan University, Chengdu, Sichuan 610041, China

[5] Department of Laboratory Medicine, West China Hospital, Sichuan University, Chengdu, Sichuan 610041, China

[6] Key Laboratory of Drug-Targeting and Drug Delivery System of the Education Ministry, Sichuan Engineering Laboratory for Plant-Sourced Drug and Sichuan Research Center for Drug Precision Industrial Technology, West China School of Pharmacy, Sichuan University, Chengdu, Sichuan 610041, China

*** Correspondence**:
Xiao-Jun Lu
luxiaojun1972@163.com
Zhi-Yao He
heyaode@163.com, zhiyaohe@scu.edu.cn
[†] **These authors have contributed equally to this work.**

Keywords: azathioprine, ITPA, TPMT, NUDT15, myelosuppression, adverse drug reaction

Supplementary materials

Supplement Figure legends

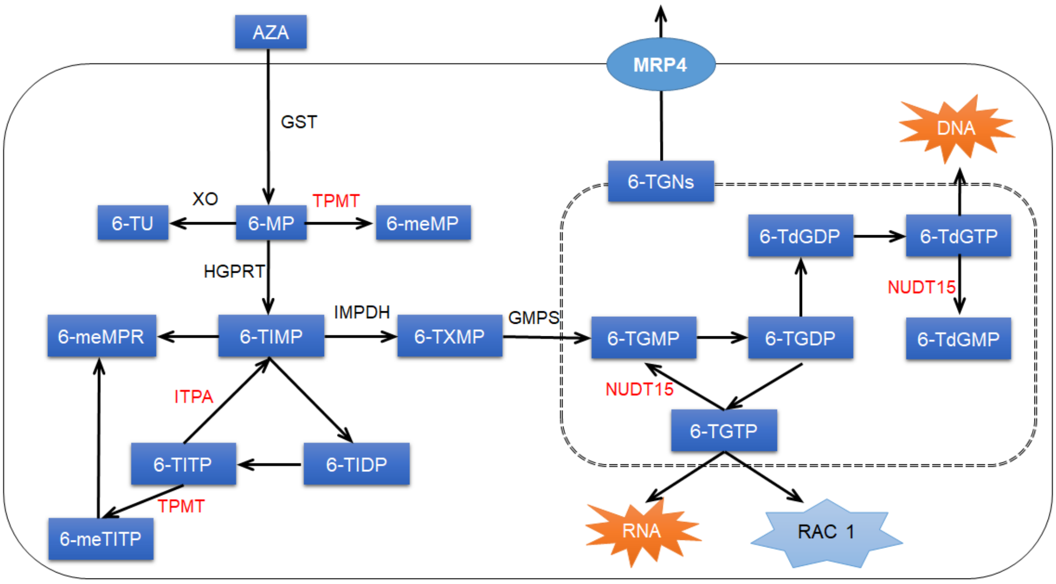

Supplement Figure S1. Azathioprine metabolism process. 6-MP, 6-mercaptopurine; 6-TU, 6-thiouric acid; 6-meMP, 6-methylmercaptopurine; 6-TIMP, 6-thioinosine monophosphate; 6-TIDP, 6-thioinosine diphosphate; 6-TITP, 6-thioinosine triphosphate; 6-MeTITP, 6-methylthioinosine triphosphate; 6-meMPR, 6-methylmercaptopurine ribonucleotides; 6-TXMP, 6-thioxanthosine monophosphate; 6-TGMP, 6-tioguanine monophosphate; 6-TGDP, 6-tioguanine diphosphate; 6-TGTP, 6-tioguanine triphosphate; 6-TdGDP, 6-thiodeoxy guanosine diphosphate; 6-TdGTP, 6-thiodeoxy guanosine triphosphate; 6-TdGMP, 6-thiodeoxy guanosine monophosphate; 6-TGNs, 6-tioguanine nucleotides; GST, glutathione S-transferase; XO, xanthine oxidase; *TPMT*, thiopurine S-methyltransferase; HGPRT, hypoxanthine-guanine phosphoribosyl transferase; *ITPA*, inosine triphosphate pyrophosphatase; IMPDH, inosine monophosphate dehydrogenase; GMPS, guanosine monophosphate synthetase; *NUDT15*, nucleoside diphosphate-liked moiety X motif 15; MRP4, multi-drug resistance protein 4.

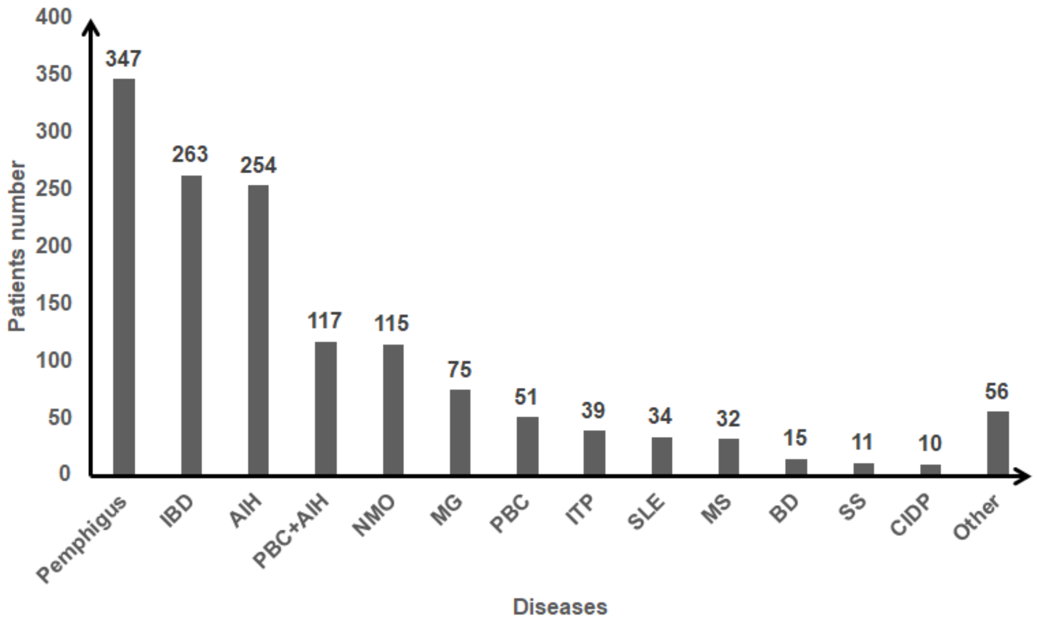

Supplement Figure S2. Number of patients with each disease. IBD, inflammatory bowel disease; AIH, autoimmune hepatitis; NMO, neuromyelitis optica; MG, myasthenia gravis; PBC, primary biliary cirrhosis; ITP, idiopathic thrombocytopenic purpura; SLE, systemic lupus erythematosus; MS, multiple sclerosis; BD, behcet disease; SS, sjogren syndrome; CIDP, chronic inflammatory demyelinating polyneuropathy.

Supplement Tables

Supplement Table S1. Genotypes of all patients (please see the separate Excel file)

Supplement Table S2 Incidence of myelosuppression in patients with *ITPA* (94C>A), *TPMT*3* (T>C), and *NUDT15* (415C>T) genotypes

		**Genotype**		**P-value**
ITPA (94C>A)	AA	AC	CC	
Incidence	2/8 (25.00%)	14/119 (11.76%)	32/389 (8.22%)	0.155
Grade	II-III	I-IV	I-IV	
[a] WBC count, $\times 10^9$/L	1.97 ± 0.15	2.72 ± 0.73	2.50 ± 0.91	0.456
MDD of AZA (mg)	70.88 ± 24.79	61.10 ± 21.73	71.65 ± 29.46	0.002
*TPMT*3* (T>C)	CC	TC	TT	
Incidence	0/1	4/20 (20.00%)	50/561 (8.91%)	0.188
Grade	-	II-IV	I-IV	
[a] WBC count, $\times 10^9$/L	-	1.84 ± 1.22	2.56 ± 0.84	0.264
MDD of AZA (mg)	67.00	66.30 ± 27.73	69.32 ± 28.17	0.892
NUDT15 (415C>T)	TT	CT	CC	
Incidence	5/6 (83.33%)	12/102 (11.76%)	37/474 (7.81%)	0.008×10^{-3}
Grade	I, IV	I-IV	I-IV	
[a] WBC count, $\times 10^9$/L	1.36 ± 1.59	2.32 ± 0.73	2.72 ± 0.67	0.002
MDD of AZA (mg)	77.08 ± 66.34	61.65 ± 27.13	70.73 ± 27.41	0.010

Data are n (%) or mean ± standard deviation. [a] WBC count: data from patients with myelosuppression.
