# Supplementary material for: Association Between Genetic Polymorphisms of Metabolic Enzymes and Azathioprine-Induced Myelosuppression in 1,419 Chinese Patients: A Retrospective Study
Source: Front Pharmacol. 2021 May 18;12:672769. doi: 10.3389/fphar.2021.672769 (PMC8167793; doi:10.3389/fphar.2021.672769)
Supplement: Supplementary file 2 [file DataSheet1.pdf]

Genotypes of all patients			
Case number	*ITPA* (94C>A) genotype	*TPMT*3* (T>C) genotype	*NUDT15* (415C>T) genotype
1	AA	TC	CC
2	AA	TC	CC
3	AA	TT	CC
4	AA	TT	CC
5	AA	TT	CC
6	AA	TT	CC
7	AA	TT	CC
8	AA	TT	CC
9	AA	TT	CC
10	AA	TT	CC
11	AA	TT	CC
12	AA	TT	CC
13	AA	TT	CC
14	AA	TT	CC
15	AA	TT	CC
16	AA	TT	CC
17	AA	TT	CC
18	AA	TT	CC
19	AA	TT	CC
20	AA	TT	CC
21	AA	TT	CC
22	AA	TT	CC
23	AA	TT	CC
24	AA	TT	CC
25	AA	TT	CC
26	AA	TT	CC
27	/	TT	TT
28	/	TC	TT
29	AA	TT	TT
30	CA	TT	TT
31	CA	TT	TT
32	CA	TT	TT
33	CA	TT	TT
34	CA	TT	TT
35	CA	TT	TT
36	CA	TT	CC
37	CA	TT	CC
38	CA	TC	CC
39	CA	TT	CC
40	CA	TT	CC
41	CA	TT	CC
42	CA	TT	CC
43	CA	TT	CC
44	CA	TT	CC
45	CA	TT	CC
46	CA	TT	CC
47	CA	TT	CC
48	CA	TT	CC
49	CA	TT	CC
50	CA	TT	CC

Genotypes of all patients			
51	CA	TT	CC
52	CA	TT	CC
53	CA	TT	CC
54	CA	TT	CC
55	CA	TT	CC
56	CA	TT	CC
57	CA	TT	CC
58	CA	TT	CC
59	CA	TT	CC
60	CA	TT	CC
61	CA	TT	CC
62	CA	TT	CC
63	CA	TT	CC
64	CA	TT	CC
65	CA	TT	CC
66	CA	TT	CC
67	CA	TT	CC
68	CA	TT	CC
69	CA	TT	CC
70	CA	TT	CC
71	CA	TT	CC
72	CA	TT	CC
73	CA	TT	CC
74	CA	TT	CC
75	CA	TT	CC
76	CA	TT	CC
77	CA	TT	CC
78	CA	TT	CC
79	CA	TT	CC
80	CA	TT	CC
81	CA	TT	CC
82	CA	TT	CC
83	CA	TT	CC
84	CA	TT	CC
85	CA	TT	CC
86	CA	TT	CC
87	CA	TT	CC
88	CA	TT	CC
89	CA	TT	CC
90	CA	TT	CC
91	CA	TT	CC
92	CA	TT	CC
93	CA	TT	CC
94	CA	TT	CC
95	CA	TT	CC
96	CA	TT	CC
97	CA	TT	CC
98	CA	TT	CC
99	CA	TT	CC
100	CA	TT	CC
101	CA	TC	CC
102	CA	TT	CC

Genotypes of all patients			
103	CA	TT	CC
104	CA	TT	CC
105	CA	TT	CC
106	CA	TT	CC
107	CA	TT	CC
108	CA	TT	CC
109	CA	TT	CC
110	CA	TT	CC
111	CA	TT	CC
112	CA	TT	CC
113	CA	TT	CC
114	CA	TT	CC
115	CA	TC	CC
116	CA	TT	CC
117	CA	TT	CC
118	CA	TT	CC
119	CA	TT	CC
120	CA	TT	CC
121	CA	TT	CC
122	CA	TT	CC
123	CA	TT	CC
124	CA	TT	CC
125	CA	TT	CC
126	CA	TC	CC
127	CA	TT	CC
128	CA	TT	CC
129	CA	TT	CC
130	CA	TT	CC
131	CA	TT	CC
132	CA	TC	CC
133	CA	TT	CC
134	CA	TT	CC
135	CA	TT	CC
136	CA	TT	CC
137	CA	TT	CC
138	CA	TT	CC
139	CA	TT	CC
140	CA	TT	CC
141	CA	TC	CC
142	CA	TT	CC
143	CA	TT	CC
144	CA	TT	CC
145	CA	TT	CC
146	CA	TC	CC
147	CA	TT	CC
148	CA	TT	CC
149	CA	TT	CC
150	CA	TT	CC
151	CA	TT	CC
152	CA	TT	CC
153	CA	TT	CC
154	CA	TT	CC

Genotypes of all patients			
155	CA	TT	CC
156	CA	TT	CC
157	CA	TT	CC
158	CA	TT	CC
159	CA	TT	CC
160	CA	TT	CC
161	CA	TT	CC
162	CA	TT	CC
163	CA	TT	CC
164	CA	TT	CC
165	CA	TT	CC
166	CA	TT	CC
167	CA	TC	CC
168	CA	TT	CC
169	CA	TT	CC
170	CA	TT	CC
171	CA	TT	CC
172	CA	TT	CC
173	CA	TT	CC
174	CA	TT	CC
175	CA	TT	CC
176	CA	TT	CC
177	CA	TT	CC
178	CA	TT	CC
179	CA	TT	CC
180	CA	TT	CC
181	CA	TT	CC
182	CA	TT	CC
183	CA	TT	CC
184	CA	TT	CC
185	CA	TT	CC
186	CA	TT	CC
187	CA	TT	CC
188	CA	TT	CC
189	CA	TT	CC
190	CA	TT	CC
191	CA	TT	CC
192	CA	TT	CC
193	CA	TT	CC
194	CA	TT	CC
195	CA	TC	CC
196	CA	TT	CC
197	CA	TT	CC
198	CA	TT	CC
199	CA	TT	CC
200	CA	TT	CC
201	CA	TT	CC
202	CA	TT	CC
203	CA	TT	CC
204	CA	TT	CC
205	CA	TT	CC
206	CA	TT	CC

Genotypes of all patients			
207	CA	TT	CC
208	CA	TT	CC
209	CA	TT	CC
210	CA	TT	CC
211	CA	TT	CC
212	CA	TT	CC
213	CA	TT	CC
214	CA	TT	CC
215	CA	TT	CC
216	CA	TT	CC
217	CA	TT	CC
218	CA	TT	CC
219	CA	TT	CC
220	CA	TT	CC
221	CA	TT	CC
222	CA	TT	CC
223	CA	TT	CC
224	CA	TT	CC
225	CA	TT	CC
226	CA	TT	CC
227	CA	TT	CC
228	CA	TT	CC
229	CA	TT	CC
230	CA	TT	CC
231	CA	TT	CC
232	CA	TT	CC
233	CA	TT	CC
234	CA	TT	CC
235	CA	TT	CC
236	CA	TT	CC
237	CA	TT	CC
238	CA	TT	CC
239	CA	TT	CC
240	CA	TT	CC
241	CA	TT	CC
242	CA	TT	CC
243	CA	TT	CC
244	CA	TT	CC
245	CA	TT	CC
246	CA	TT	CC
247	CA	TT	CC
248	CA	TT	CC
249	CA	TT	CC
250	CA	TT	CC
251	CA	TT	CC
252	CA	TC	CC
253	CA	TT	CC
254	CA	TT	CC
255	CA	TT	CC
256	CA	TT	CC
257	CA	TT	CC
258	CA	TT	CC

**Genotypes of all patients**			
259	CA	TT	CC
260	CA	TT	CC
261	CA	TT	CC
262	CA	TT	CC
263	CA	TT	CC
264	CA	TT	CC
265	CA	TT	CC
266	CA	TT	CC
267	CA	TT	CC
268	CA	TT	CC
269	CA	TT	CC
270	CA	TT	CC
271	CA	TT	CC
272	CA	TT	CC
273	CA	TT	CC
274	CA	TT	CC
275	CA	TT	CC
276	CA	TT	CC
277	CA	TT	CC
278	CA	TT	CC
279	CA	TT	CC
280	CA	TT	CC
281	CA	TT	CC
282	CA	TT	CC
283	CA	TT	CC
284	CA	TT	CC
285	CA	TT	CC
286	CA	TT	CC
287	CA	TT	CC
288	CA	TT	TT
289	CA	TT	TT
290	CA	TT	TT
291	CA	TT	TT
292	**CC**	**TT**	**TT**
293	**CC**	**TT**	**TT**
294	**CC**	**TT**	**TT**
295	**CC**	**TT**	**TT**
296	**CC**	**TT**	**TT**
297	**CC**	**TT**	**TT**
298	**CC**	**TT**	**TT**
299	**CC**	**TT**	**TT**
300	**CC**	**TT**	**TT**
301	**CC**	**TT**	**TT**
302	**CC**	**TT**	**TT**
303	**CC**	**TT**	**TT**
304	**CC**	**TT**	**TT**
305	**CC**	**TT**	**TT**
306	**CC**	**TT**	**TT**
307	/	**TT**	**CT**
308	/	**TT**	**CT**
309	/	**TT**	**CT**
310	/	**TT**	**CT**

Genotypes of all patients			
311	/	**TT**	**CT**
312	/	**TT**	**CT**
313	/	**TT**	**CT**
314	/	**TT**	**CT**
315	/	**TT**	**CT**
316	/	**TT**	**CT**
317	/	**TT**	**CT**
318	/	**TT**	**CT**
319	/	**TT**	**CT**
320	/	**TT**	**CT**
321	/	**TT**	**CT**
322	/	**TT**	**CT**
323	/	**TT**	**CT**
324	/	**TT**	**CT**
325	/	**TT**	**CT**
326	/	**TT**	**CT**
327	/	**TT**	**CT**
328	/	**TT**	**CT**
329	/	**TT**	**CT**
330	/	**TT**	**CT**
331	/	**TT**	**CT**
332	/	**TT**	**CT**
333	/	**TT**	**CT**
334	**AA**	**TT**	**CT**
335	**AA**	**TT**	**CT**
336	**AA**	**TT**	**CT**
337	**AA**	**TT**	**CT**
338	**AA**	**TT**	**CT**
339	**AA**	**TT**	**CT**
340	**AA**	**TT**	**CT**
341	**AA**	**TT**	**CT**
342	CA	TT	CT
343	CA	TT	CT
344	CA	TT	CT
345	CA	TT	CT
346	CA	TT	CT
347	CA	TT	CT
348	CA	TT	CT
349	CA	TT	CT
350	CA	TT	CT
351	CA	TT	CT
352	CA	TT	CT
353	CA	TT	CT
354	CA	TT	CT
355	CA	TT	CT
356	CA	TT	CT
357	CA	TT	CT
358	CA	TT	CT
359	CA	TT	CT
360	CA	TT	CT
361	CA	TT	CT
362	CA	TT	CT

Genotypes of all patients			
363	CA	TT	CT
364	CA	TT	CT
365	CA	TT	CT
366	CA	TT	CT
367	CA	TT	CT
368	CA	TT	CT
369	CA	TT	CT
370	CA	TT	CT
371	CA	TT	CT
372	CA	TT	CT
373	CA	TT	CT
374	CA	TT	CT
375	CA	TT	CT
376	CC	CC	CC
377	CC	CC	CC
378	CC	TC	CC
379	CC	TC	CC
380	CC	TC	CC
381	CC	TT	CC
382	CC	TT	CC
383	CC	TT	CC
384	CC	TT	CC
385	CC	TT	CC
386	CC	TT	CC
387	CC	TT	CC
388	CC	TT	CC
389	CC	TT	CC
390	CC	TT	CC
391	CC	TT	CC
392	CC	TT	CC
393	CC	TT	CC
394	CC	TT	CC
395	CC	TT	CC
396	CC	TT	CC
397	CC	TT	CC
398	CC	TT	CC
399	CC	TT	CC
400	CC	TT	CC
401	CC	TT	CC
402	CC	TT	CC
403	CC	TT	CC
404	CC	TT	CC
405	CC	TT	CC
406	CC	TT	CC
407	CC	TT	CC
408	CC	TT	CC
409	CC	TT	CC
410	CC	TT	CC
411	CC	TT	CC
412	CC	TT	CC
413	CC	TT	CC
414	CC	TT	CC

Genotypes of all patients			
415	CC	TT	CC
416	CC	TT	CC
417	CC	TT	CC
418	CC	TT	CC
419	CC	TT	CC
420	CC	TT	CC
421	CC	TT	CC
422	CC	TT	CC
423	CC	TT	CC
424	CC	TT	CC
425	CC	TC	CC
426	CC	TT	CC
427	CC	TT	CC
428	CC	TT	CC
429	CC	TT	CC
430	CC	TT	CC
431	CC	TT	CC
432	CC	TT	CC
433	CC	TT	CC
434	CC	TT	CC
435	CC	TT	CC
436	CC	TT	CC
437	CC	TT	CC
438	CC	TT	CC
439	CC	TT	CC
440	CC	TT	CC
441	CC	TT	CC
442	CC	TT	CC
443	CC	TT	CC
444	CC	TT	CC
445	CC	TT	CC
446	CC	TC	CC
447	CC	TT	CC
448	CC	TC	CC
449	CC	TT	CC
450	CC	TT	CC
451	CC	TT	CC
452	CC	TT	CC
453	CC	TT	CC
454	CC	TT	CC
455	CC	TT	CC
456	CC	TT	CC
457	CC	TT	CC
458	CC	TT	CC
459	CC	TT	CC
460	CC	TT	CC
461	CC	TT	CC
462	CC	TT	CC
463	CC	TC	CC
464	CC	TT	CC
465	CC	TT	CC
466	CC	TT	CC

Genotypes of all patients			
467	CC	TT	CC
468	CC	TT	CC
469	CC	TT	CC
470	CC	TT	CC
471	CC	TT	CC
472	CC	TT	CC
473	CC	TT	CC
474	CC	TT	CC
475	CC	TT	CC
476	CC	TT	CC
477	CC	TT	CC
478	CC	TT	CC
479	CC	TT	CC
480	CC	TT	CC
481	CC	TT	CC
482	CC	TT	CC
483	CC	TT	CC
484	CC	TT	CC
485	CC	TT	CC
486	CC	TT	CC
487	CC	TT	CC
488	CC	TT	CC
489	CC	TT	CC
490	CC	TT	CC
491	CC	TT	CC
492	CC	TT	CC
493	CC	TT	CC
494	CC	TT	CC
495	CC	TT	CC
496	CC	TT	CC
497	CC	TT	CC
498	CC	TT	CC
499	CC	TT	CC
500	CC	TT	CC
501	CC	TT	CC
502	CC	TT	CC
503	CC	TT	CC
504	CC	TT	CC
505	CC	TT	CC
506	CC	TT	CC
507	CC	TT	CC
508	CC	TT	CC
509	CC	TT	CC
510	CC	TT	CC
511	CC	TC	CC
512	CC	TT	CC
513	CC	TT	CC
514	CC	TT	CC
515	CC	TT	CC
516	CC	TT	CC
517	CC	TT	CC
518	CC	TT	CC

Genotypes of all patients			
519	CC	TT	CC
520	CC	TT	CC
521	CC	TT	CC
522	CC	TT	CC
523	CC	TT	CC
524	CC	TT	CC
525	CC	TT	CC
526	CC	TT	CC
527	CC	TT	CC
528	CC	TT	CC
529	CC	TT	CC
530	CC	TT	CC
531	CC	TT	CC
532	CC	TT	CC
533	CC	TT	CC
534	CC	TT	CC
535	CC	TT	CC
536	CC	TT	CC
537	CC	TT	CC
538	CC	TC	CC
539	CC	TT	CC
540	CC	TT	CC
541	CC	TT	CC
542	CC	TT	CC
543	CC	TT	CC
544	CC	TT	CC
545	CC	TT	CC
546	CC	TT	CC
547	CC	TC	CC
548	CC	TT	CC
549	CC	TT	CC
550	CC	TT	CC
551	CC	TT	CC
552	CC	TT	CC
553	CC	TT	CC
554	CC	TT	CC
555	CC	TC	CC
556	CC	TT	CC
557	CC	TT	CC
558	CC	TT	CC
559	CC	TT	CC
560	CC	TT	CC
561	CC	TT	CC
562	CC	TT	CC
563	CC	TT	CC
564	CC	TT	CC
565	CC	TT	CC
566	CC	TT	CC
567	CC	TC	CC
568	CC	TT	CC
569	CC	TT	CC
570	CC	TT	CC

Genotypes of all patients			
571	CC	TT	CC
572	CC	TT	CC
573	CC	TT	CC
574	CC	TT	CC
575	CC	TT	CC
576	CC	TT	CC
577	CC	TT	CC
578	CC	TT	CC
579	CC	TT	CC
580	CC	TT	CC
581	CC	TT	CC
582	CC	TT	CC
583	CC	TT	CC
584	CC	TT	CC
585	CC	TC	CC
586	CC	TT	CC
587	CC	TT	CC
588	CC	TT	CC
589	CC	TT	CC
590	CC	TT	CC
591	CC	TT	CC
592	CC	TT	CC
593	CC	TT	CC
594	CC	TT	CC
595	CC	TT	CC
596	CC	TT	CC
597	CC	TT	CC
598	CC	TT	CC
599	CC	TT	CC
600	CC	TT	CC
601	CC	TT	CC
602	CC	TT	CC
603	CC	TT	CC
604	CC	TT	CC
605	CC	TC	CC
606	CC	TT	CC
607	CC	TT	CC
608	CC	TT	CC
609	CC	TT	CC
610	CC	TT	CC
611	CC	TT	CC
612	CC	TT	CC
613	CC	TT	CC
614	CC	TT	CC
615	CC	TT	CC
616	CC	TT	CC
617	CC	TT	CC
618	CC	TT	CC
619	CC	TT	CC
620	CC	TT	CC
621	CC	TT	CC
622	CC	TT	CC

Genotypes of all patients			
623	CC	TT	CC
624	CC	TT	CC
625	CC	TT	CC
626	CC	TT	CC
627	CC	TT	CC
628	CC	TT	CC
629	CC	TT	CC
630	CC	TT	CC
631	CC	TT	CC
632	CC	TT	CC
633	CC	TT	CC
634	CC	TT	CC
635	CC	TT	CC
636	CC	TT	CC
637	CC	TT	CC
638	CC	TT	CC
639	CC	TT	CC
640	CC	TT	CC
641	CC	TT	CC
642	CC	TT	CC
643	CC	TT	CC
644	CC	TT	CC
645	CC	TT	CC
646	CC	TT	CC
647	CC	TT	CC
648	CC	TT	CC
649	CC	TT	CC
650	CC	TT	CC
651	CC	TT	CC
652	CC	TT	CC
653	CC	TT	CC
654	CC	TT	CC
655	CC	TT	CC
656	CC	TT	CC
657	CC	TT	CC
658	CC	TT	CC
659	CC	TT	CC
660	CC	TT	CC
661	CC	TT	CC
662	CC	TT	CC
663	CC	TT	CC
664	CC	TC	CC
665	CC	TT	CC
666	CC	TT	CC
667	CC	TT	CC
668	CC	TC	CC
669	CC	TT	CC
670	CC	TT	CC
671	CC	TT	CC
672	CC	TT	CC
673	CC	TT	CC
674	CC	TT	CC

Genotypes of all patients			
675	CC	TT	CC
676	CC	TT	CC
677	CC	TT	CC
678	CC	TC	CC
679	CC	TT	CC
680	CC	TT	CC
681	CC	TT	CC
682	CC	TT	CC
683	CC	TT	CC
684	CC	TT	CC
685	CC	TT	CC
686	CC	TT	CC
687	CC	TT	CC
688	CC	TT	CC
689	CC	TT	CC
690	CC	TT	CC
691	CC	TT	CC
692	CC	TT	CC
693	CC	TT	CC
694	CC	TT	CC
695	CC	TT	CC
696	CC	TT	CC
697	CC	TT	CC
698	CC	TT	CC
699	CC	TT	CC
700	CC	TT	CC
701	CC	TT	CC
702	CC	TT	CC
703	CC	TT	CC
704	CC	TT	CC
705	CC	TT	CC
706	CC	TT	CC
707	CC	TT	CC
708	CC	TT	CC
709	CC	TT	CC
710	CC	TT	CC
711	CC	TT	CC
712	CC	TT	CC
713	CC	TT	CC
714	CC	TT	CC
715	CC	TT	CC
716	CC	TT	CC
717	CC	TT	CC
718	CC	TT	CC
719	CC	TT	CC
720	CC	TT	CC
721	CC	TT	CC
722	CC	TT	CC
723	CC	TT	CC
724	CC	TT	CC
725	CC	TT	CC
726	CC	TT	CC

Genotypes of all patients			
727	CC	TT	CC
728	CC	TT	CC
729	CC	TT	CC
730	CC	TT	CC
731	CC	TT	CC
732	CC	TT	CC
733	CC	TT	CC
734	CC	TT	CC
735	CC	TT	CC
736	CC	TT	CC
737	CC	TT	CC
738	CC	TT	CC
739	CC	TT	CC
740	CC	TT	CC
741	CC	TT	CC
742	CC	TT	CC
743	CC	TT	CC
744	CC	TT	CC
745	CC	TT	CC
746	CC	TT	CC
747	CC	TT	CC
748	CC	TT	CC
749	CC	TT	CC
750	CC	TT	CC
751	CC	TT	CC
752	CC	TT	CC
753	CC	TT	CC
754	CC	TT	CC
755	CC	TT	CC
756	CC	TT	CC
757	CC	TT	CC
758	CC	TT	CC
759	CC	TT	CC
760	CC	TT	CC
761	CC	TT	CC
762	CC	TT	CC
763	CC	TT	CC
764	CC	TT	CC
765	CC	TT	CC
766	CC	TT	CC
767	CC	TT	CC
768	CC	TT	CC
769	CC	TT	CC
770	CC	TT	CC
771	CC	TT	CC
772	CC	TT	CC
773	CC	TT	CC
774	CC	TT	CC
775	CC	TT	CC
776	CC	TT	CC
777	CC	TT	CC
778	CC	TT	CC

Genotypes of all patients			
779	CC	TT	CC
780	CC	TT	CC
781	CC	TT	CC
782	CC	TT	CC
783	CC	TT	CC
784	CC	TT	CC
785	CC	TT	CC
786	CC	TT	CC
787	CC	TT	CC
788	CC	TT	CC
789	CC	TT	CC
790	CC	TT	CC
791	CC	TT	CC
792	CC	TT	CC
793	CC	TT	CC
794	CC	TT	CC
795	CC	TT	CC
796	CC	TT	CC
797	CC	TT	CC
798	CC	TT	CC
799	CC	TT	CC
800	CC	TT	CC
801	CC	TT	CC
802	CC	TT	CC
803	CC	TT	CC
804	CC	TT	CC
805	CC	TT	CC
806	CC	TT	CC
807	CC	TT	CC
808	CC	TT	CC
809	CC	TT	CC
810	CC	TT	CC
811	CC	TT	CC
812	CC	TT	CC
813	CC	TC	CC
814	CC	TC	CC
815	CC	TT	CC
816	CC	TT	CC
817	CC	TT	CC
818	CC	TT	CC
819	CC	TT	CC
820	CC	TT	CC
821	CC	TT	CC
822	CC	TT	CC
823	CC	TT	CC
824	CC	TT	CC
825	CC	TT	CC
826	CC	TT	CC
827	CC	TT	CC
828	CC	TT	CC
829	CC	TT	CC
830	CC	TT	CC

Genotypes of all patients			
831	CC	TT	CC
832	CC	TT	CC
833	CC	TT	CC
834	CC	TT	CC
835	CC	TT	CC
836	CC	TT	CC
837	CC	TT	CC
838	CC	TT	CC
839	CC	TT	CC
840	CC	TT	CC
841	CC	TT	CC
842	CC	TT	CC
843	CC	TT	CC
844	CC	TT	CC
845	CC	TT	CC
846	CC	TT	CC
847	CC	TT	CC
848	CC	TT	CC
849	CC	TT	CC
850	CC	TT	CC
851	CC	TT	CC
852	CC	TT	CC
853	CC	TT	CC
854	CC	TT	CC
855	CC	TT	CC
856	CC	TT	CC
857	CC	TT	CC
858	CC	TT	CC
859	CC	TT	CC
860	CC	TT	CC
861	CC	TT	CC
862	CC	TT	CC
863	CC	TT	CC
864	CC	TT	CC
865	CC	TT	CC
866	CC	TT	CC
867	CC	TT	CC
868	CC	TT	CC
869	CC	TT	CC
870	CC	TT	CC
871	CC	TT	CC
872	CC	TT	CC
873	CC	TT	CC
874	CC	TT	CC
875	CC	TT	CC
876	CC	TT	CC
877	CC	TT	CC
878	CC	TT	CC
879	CC	TT	CC
880	CC	TT	CC
881	CC	TT	CC
882	CC	TT	CC
883	CC	TC	CC

Genotypes of all patients			
884	CC	TT	CC
885	CC	TT	CC
886	CC	TT	CC
887	CC	TT	CC
888	CC	TT	CC
889	CC	TT	CC
890	CC	TT	CC
891	CC	TT	CC
892	CC	TT	CC
893	CC	TT	CC
894	CC	TT	CC
895	CC	TT	CC
896	CC	TT	CC
897	CC	TT	CC
898	CC	TT	CC
899	CC	TT	CC
900	CC	TT	CC
901	CC	TT	CC
902	CC	TT	CC
903	CC	TT	CC
904	CC	TT	CC
905	CC	TT	CC
906	CC	TT	CC
907	CC	TT	CC
908	CC	TT	CC
909	CC	TT	CC
910	CC	TT	CC
911	CC	TT	CC
912	CC	TT	CC
913	CC	TT	CC
914	CC	TT	CC
915	CC	TT	CC
916	CC	TT	CC
917	CC	TT	CC
918	CC	TT	CC
919	CC	TT	CC
920	CC	TT	CC
921	CC	TT	CC
922	CC	TT	CC
923	CC	TT	CC
924	CC	TT	CC
925	CC	TT	CC
926	CC	TT	CC
927	CC	TT	CC
928	CC	TT	CC
929	CC	TT	CC
930	CC	TT	CC
931	CC	TT	CC
932	CC	TT	CC
933	CC	TT	CC
934	CC	TT	CC
935	CC	TT	CC
936	CC	TT	CC
937	CC	TT	CC
938	CC	TT	CC
939	CC	TT	CC
940	CC	TT	CC
941	CC	TT	CC
942	CC	TT	CC
943	CC	TT	CC

Genotypes of all patients			
944	CC	TT	CC
945	CC	TT	CC
946	CC	TT	CC
947	CC	TT	CC
948	CC	TT	CC
949	CC	TC	CC
950	CC	TT	CC
951	CC	TT	CC
952	CC	TT	CC
953	CC	TT	CC
954	CC	TT	CC
955	CC	TT	CC
956	CC	TT	CC
957	CC	TT	CC
958	CC	TT	CC
959	CC	TT	CC
960	CC	TT	CC
961	CC	TT	CC
962	CC	TT	CC
963	CC	TT	CC
964	CC	TT	CC
965	CC	TT	CC
966	CC	TT	CC
967	CC	TC	CC
968	CC	TT	CC
969	CC	TT	CC
970	CC	TT	CC
971	CC	TT	CC
972	CC	TT	CC
973	CC	TT	CC
974	CC	TT	CC
975	CC	TT	CC
976	CC	TT	CC
977	CC	TT	CC
978	CC	TT	CC
979	CC	TT	CC
980	CC	TT	CC
981	CC	TT	CC
982	CC	TT	CC
983	CC	TT	CC
984	CC	TC	CC
985	CC	TT	CC
986	CC	TT	CC
987	CC	TT	CC
988	CC	TT	CC
989	CC	TT	CC
990	CC	TT	CC
991	CC	TT	CC
992	CC	TT	CC
993	CC	TT	CC
994	CC	TT	CC
995	CC	TT	CC
996	CC	TT	CC
997	CC	TT	CC
998	CC	TT	CC
999	CC	TT	CC
1000	CC	TT	CC
1001	CC	TT	CC
1002	CC	TT	CC
1003	CC	TT	CC

Genotypes of all patients			
1004	CC	TT	CC
1005	CC	TT	CC
1006	CC	TT	CC
1007	CC	TT	CC
1008	CC	TT	CC
1009	CC	TT	CC
1010	CC	TT	CC
1011	CC	TT	CC
1012	CC	TT	CC
1013	CC	TT	CC
1014	CC	TT	CC
1015	CC	TT	CC
1016	CC	TT	CC
1017	CC	TT	CC
1018	CC	TT	CC
1019	CC	TT	CC
1020	CC	TT	CC
1021	CC	TT	CC
1022	CC	TT	CC
1023	CC	TT	CC
1024	CC	TT	CC
1025	CC	TT	CC
1026	CC	TT	CC
1027	CC	TT	CC
1028	CC	TT	CC
1029	CC	TT	CC
1030	CC	TT	CC
1031	CC	TT	CC
1032	CC	TT	CC
1033	CC	TT	CC
1034	CC	TT	CC
1035	CC	TT	CC
1036	CC	TT	CC
1037	CC	TT	CC
1038	CC	TT	CC
1039	CC	TT	CC
1040	CC	TT	CC
1041	CC	TT	CC
1042	CC	TT	CC
1043	CC	TT	CC
1044	CC	TT	CC
1045	CC	TT	CC
1046	CC	TT	CC
1047	CC	TT	CC
1048	CC	TT	CC
1049	CC	TT	CC
1050	CC	TT	CC
1051	CC	TT	CC
1052	CC	TT	CC
1053	CC	TT	CC
1054	CC	TT	CC
1055	CC	TT	CC
1056	CC	TT	CC
1057	CC	TT	CC
1058	CC	TT	CC
1059	CC	TT	CC
1060	CC	TT	CC
1061	CC	TT	CC
1062	CC	TT	CC
1063	CC	TT	CC

Genotypes of all patients			
1064	CC	TT	CC
1065	CC	TT	CC
1066	CC	TT	CC
1067	CC	TT	CC
1068	CC	TT	CC
1069	CC	TT	CC
1070	CC	TT	CC
1071	CC	TT	CC
1072	CC	TT	CC
1073	CC	TT	CC
1074	CC	TT	CC
1075	CC	TT	CC
1076	CC	TT	CC
1077	CC	TT	CC
1078	CC	TT	CC
1079	CC	TT	CC
1080	CC	TT	CC
1081	CC	TT	CC
1082	CC	TT	CC
1083	CC	TT	CC
1084	CC	TT	CC
1085	CC	TT	CC
1086	CC	TT	CC
1087	CC	TT	CC
1088	CC	TT	CC
1089	CC	TT	CC
1090	CC	TT	CC
1091	CC	TT	CC
1092	CC	TT	CC
1093	CC	TT	CC
1094	CC	TT	CC
1095	CC	TT	CC
1096	CA	TT	CT
1097	CA	TT	CT
1098	CA	TT	CT
1099	CA	TT	CT
1100	CA	TT	CT
1101	CA	TT	CT
1102	CA	TT	CT
1103	CA	TT	CT
1104	CA	TT	CT
1105	CA	TT	CT
1106	CA	TT	CT
1107	CA	TT	CT
1108	CA	TC	CT
1109	CA	TT	CT
1110	CA	TT	CT
1111	CA	TT	CT
1112	CA	TT	CT
1113	CA	TT	CT
1114	CA	TT	CT
1115	CA	TT	CT
1116	CA	TT	CT
1117	CA	TT	CT
1118	CA	TT	CT
1119	CA	TT	CT
1120	CA	TT	CT
1121	CA	TT	CT
1122	CA	TT	CT
1123	CA	TT	CT

Genotypes of all patients			
1124	CA	TT	CT
1125	CA	TT	CT
1126	CA	TT	CT
1127	CA	TT	CT
1128	CA	TT	CT
1129	CA	TT	CT
1130	CA	TT	CT
1131	CA	TT	CT
1132	CA	TT	CT
1133	CA	TT	CT
1134	CA	TT	CT
1135	CA	TT	CT
1136	CA	TT	CT
1137	CA	TT	CT
1138	CA	TT	CT
1139	CA	TT	CT
1140	CC	TC	CT
1141	CC	TT	CT
1142	CC	TT	CT
1143	CC	TT	CT
1144	CC	TT	CT
1145	CC	TT	CT
1146	CC	TT	CT
1147	CC	TT	CT
1148	CC	TT	CT
1149	CC	TT	CT
1150	CC	TT	CT
1151	CC	TT	CT
1152	CC	TT	CT
1153	CC	TT	CT
1154	CC	TT	CT
1155	CC	TT	CT
1156	CC	TT	CT
1157	CC	TT	CT
1158	CC	TT	CT
1159	CC	TT	CT
1160	CC	TT	CT
1161	CC	TT	CT
1162	CC	TT	CT
1163	CC	TC	CT
1164	CC	TT	CT
1165	CC	TT	CT
1166	CC	TT	CT
1167	CC	TT	CT
1168	CC	TT	CT
1169	CC	TT	CT
1170	CC	TT	CT
1171	CC	TT	CT
1172	CC	TT	CT
1173	CC	TT	CT
1174	CC	TT	CT
1175	CC	TT	CT
1176	CC	TT	CT
1177	CC	TT	CT
1178	CC	TT	CT
1179	CC	TT	CT
1180	CC	TT	CT
1181	CC	TC	CT
1182	CC	TT	CT
1183	CC	TT	CT

Genotypes of all patients			
1184	CC	TC	CT
1185	CC	TT	CT
1186	CC	TT	CT
1187	CC	TT	CT
1188	CC	TT	CT
1189	CC	TT	CT
1190	CC	TT	CT
1191	CC	TT	CT
1192	CC	TT	CT
1193	CC	TT	CT
1194	CC	TT	CT
1195	CC	TT	CT
1196	CC	TC	CT
1197	CC	TT	CT
1198	CC	TT	CT
1199	CC	TT	CT
1200	CC	TT	CT
1201	CC	TT	CT
1202	CC	TT	CT
1203	CC	TT	CT
1204	CC	TT	CT
1205	CC	TT	CT
1206	CC	TT	CT
1207	CC	TT	CT
1208	CC	TT	CT
1209	CC	TT	CT
1210	CC	TT	CT
1211	CC	TT	CT
1212	CC	TT	CT
1213	CC	TT	CT
1214	CC	TT	CT
1215	CC	TT	CT
1216	CC	TT	CT
1217	CC	TT	CT
1218	CC	TT	CT
1219	CC	TT	CT
1220	CC	TT	CT
1221	CC	TT	CT
1222	CC	TT	CT
1223	CC	TT	CT
1224	CC	TC	CT
1225	CC	TT	CT
1226	CC	TT	CT
1227	CC	TT	CT
1228	CC	TT	CT
1229	CC	TT	CT
1230	CC	TT	CT
1231	CC	TT	CT
1232	CC	TT	CT
1233	CC	TT	CT
1234	CC	TT	CT
1235	CC	TT	CT
1236	CC	TT	CT
1237	CC	TT	CT
1238	CC	TT	CT
1239	CC	TT	CT
1240	CC	TT	CT
1241	CC	TT	CT
1242	CC	TT	CT
1243	CC	TT	CT

Genotypes of all patients			
1244	CC	TT	CT
1245	CC	TT	CT
1246	CC	TT	CT
1247	CC	TT	CT
1248	CC	TT	CT
1249	CC	TT	CT
1250	CC	TT	CT
1251	CC	TT	CT
1252	CC	TT	CT
1253	CC	TT	CT
1254	CC	TT	CT
1255	CC	TT	CT
1256	CC	TT	CT
1257	CC	TC	CT
1258	CC	TT	CT
1259	CC	TT	CT
1260	CC	TT	CT
1261	CC	TT	CT
1262	CC	TT	CT
1263	CC	TT	CT
1264	CC	TT	CT
1265	CC	TT	CT
1266	CC	TT	CT
1267	CC	TT	CT
1268	CC	TT	CT
1269	CC	TT	CT
1270	CC	TT	CT
1271	CC	TT	CT
1272	CC	TT	CT
1273	CC	TT	CT
1274	CC	TT	CT
1275	CC	TT	CT
1276	CC	TT	CT
1277	CC	TT	CT
1278	CC	TT	CT
1279	/	TT	CC
1280	/	TT	CC
1281	/	TT	CC
1282	/	TT	CC
1283	/	TT	CC
1284	/	TT	CC
1285	/	TT	CC
1286	/	TT	CC
1287	/	TT	CC
1288	/	TT	CC
1289	/	TT	CC
1290	/	TT	CC
1291	/	TT	CC
1292	/	TT	CC
1293	/	TT	CC
1294	/	TT	CC
1295	/	TT	CC
1296	/	TT	CC
1297	/	TT	CC
1298	/	TT	CC
1299	/	TT	CC
1300	/	TT	CC
1301	/	TT	CC
1302	/	TT	CC

Genotypes of all patients			
1303	/	TT	CC
1304	/	TT	CC
1305	/	TT	CC
1306	/	TT	CC
1307	/	TT	CC
1308	/	TT	CC
1309	/	TT	CC
1310	/	TT	CC
1311	/	TT	CC
1312	/	TT	CC
1313	/	TT	CC
1314	/	TT	CC
1315	/	TT	CC
1316	/	TT	CC
1317	/	TT	CC
1318	/	TT	CC
1319	/	TT	CC
1320	/	TT	CC
1321	/	TT	CC
1322	/	TT	CC
1323	/	TT	CC
1324	/	TT	CC
1325	/	TT	CC
1326	/	TT	CC
1327	/	TT	CC
1328	/	TT	CC
1329	/	TT	CC
1330	/	TT	CC
1331	/	TT	CC
1332	/	TT	CC
1333	/	TT	CC
1334	/	TT	CC
1335	/	TT	CC
1336	/	TT	CC
1337	/	TT	CC
1338	/	TT	CC
1339	/	TT	CC
1340	/	TT	CC
1341	/	TT	CC
1342	/	TT	CC
1343	/	TT	CC
1344	/	TT	CC
1345	/	TT	CC
1346	/	TT	CC
1347	/	TT	CC
1348	/	TT	CC
1349	/	TT	CC
1350	/	TT	CC
1351	/	TT	CC
1352	/	TT	CC
1353	/	TT	CC
1354	/	TT	CC
1355	/	TT	CC
1356	/	TT	CC
1357	/	TT	CC
1358	/	TT	CC
1359	/	TT	CC
1360	/	TT	CC
1361	/	TT	CC
1362	/	TT	CC

Genotypes of all patients			
1363	/	TT	CC
1364	/	TT	CC
1365	/	TT	CC
1366	/	TT	CC
1367	/	TT	CC
1368	/	TT	CC
1369	/	TT	CC
1370	/	TT	CC
1371	/	TT	CC
1372	/	TT	CC
1373	/	TT	CC
1374	/	TT	CC
1375	/	TT	CC
1376	/	TT	CC
1377	/	TT	CC
1378	/	TT	CC
1379	/	TT	CC
1380	/	TC	CC
1381	/	TC	CC
1382	/	TC	CC
1383	/	TC	CC
1384	/	TC	CC
1385	/	TC	CC
1386	/	TC	CC
1387	CC	TT	CT
1388	CC	TT	CT
1389	CC	TT	CT
1390	CC	TT	CT
1391	CC	TT	CT
1392	CC	TT	CT
1393	CC	TT	CT
1394	CC	TT	CT
1395	CC	TT	CT
1396	CC	TT	CT
1397	CC	TT	CT
1398	CC	TT	CT
1399	CC	TT	CT
1400	CC	TT	CT
1401	CC	TT	CT
1402	CC	TT	CT
1403	CC	TT	CT
1404	CC	TT	CT
1405	CC	TT	CT
1406	CC	TT	CT
1407	CC	TT	CT
1408	CC	TT	CT
1409	CC	TT	CT
1410	CC	TT	CT
1411	CC	TT	CT
1412	CC	TT	CT
1413	CC	TT	CT
1414	CC	TT	CT
1415	CC	TT	CT
1416	/	TT	CT
1417	/	TT	CT
1418	/	TT	CC
1419	CC	TT	CC
